# Protecting Young Lives: A Systematic Review of the Impact of Secondhand Smoke Exposure and Legislative Measures on Children's Health

**DOI:** 10.7759/cureus.72548

**Published:** 2024-10-28

**Authors:** Simret M Asfaw, Sai M Vijayawada, Yasmin Sharifian, Faiza Choudhry, Pallak Khattar, Paolo C Cavalie, Iana Malasevskaia

**Affiliations:** 1 General Practice, Haramaya University College of Health and Medical Sciences, Harar, ETH; 2 Surgery, Dr. N.T.R. University, Vijayawada, IND; 3 General Practice, RAK Medical and Health Sciences University, Ras Al Khaimah, ARE; 4 Medicine and Surgery, Peoples University of Medical and Health Sciences For Women, Nawabshah, PAK; 5 General Practice, Manipal College of Medical Sciences, Pokhara, NPL; 6 Surgery, Universidad Peruana de Ciencias Aplicadas, Lima, PER; 7 Obstetrics and Gynecology, Private Clinic of Obstetrics and Gynecology "Yana Alexandr", Sana'a, YEM; 8 Research and Development, California Institute of Behavioral Neurosciences & Psychology, Fairfield, USA

**Keywords:** behavioral issues, children's health, passive smoking, respiratory conditions, secondhand smoke, smoke-free legislation

## Abstract

Each year, a substantial number of global deaths are attributed to secondhand smoke (SHS) exposure, with children representing a significant proportion of these fatalities. This systematic review evaluates the effects of passive smoking, or SHS, on children's health outcomes, focusing on respiratory conditions, behavioral issues, cognitive impairment, growth retardation, and immune system effects. A comprehensive literature search was conducted from July 5 to July 18, 2024, across multiple databases according to the Preferred Reporting Items for Systematic Reviews and Meta-Analyses (PRISMA) 2020 guidelines. Following the set inclusion and exclusion guidelines, 12 studies were chosen for the review, and their quality was measured using the Newcastle-Ottawa Scale. The findings indicate a strong association between SHS exposure and adverse health outcomes in children, including increased rates of asthma, low birth weight, and behavioral problems. Additionally, smoke-free legislation appears to mitigate some of these harmful effects, though results vary across different regions and populations. The review underscores the urgent need for effective smoking cessation strategies and improved healthcare communication to protect children from the dangers of passive smoking.

## Introduction and background

An estimated 600,000 fatalities and about 11 million disability-adjusted life years (DALYs) occur globally each year as a result of secondhand smoke (SHS) exposure [1]. Almost 25% of these deaths and over 50% of the related DALYs are in children, and respiratory illnesses are nearly solely to blame for these outcomes [1]. Passive smoking, or SHS, is the unintentional intake of smoke by nonsmokers [2]. Regular exposure to SHS occurs for almost 40% of children in the United States, usually at the hands of a parent or caregiver [3,4].

Exposure to SHS has been thoroughly linked to detrimental health outcomes, particularly in the respiratory and cardiovascular systems [5]. In addition to other contributing reasons, children are particularly susceptible to the negative health effects of SHS because of their growing immunological and respiratory systems and quicker breathing rates [6]. Exposure to SHS has been linked to several illnesses in children, including asthma, low birth weight (LBW), otitis media (OM), lower respiratory infections (LRI), and sudden infant death syndrome (SIDS) [7].

A critical intervention to shield children and fetuses from the harmful effects of tobacco smoke exposure (TSE) is smoke-free legislation, particularly comprehensive smoke-free regulations that encompass all enclosed public spaces and workplaces [8].

This systematic review aims to evaluate the effects of passive smoking or SHS on children's health outcomes, specifically focusing on respiratory conditions, behavioral issues, cognitive impairment, growth retardation, and immune system effects. The review will also assess the short-term and long-term health impacts of children's exposure to passive smoking and the effectiveness of smoke-free legislation in reducing these harmful effects. Additionally, it seeks to understand parental preferences for smoking cessation messages and the role of healthcare communication in mitigating the risks associated with SHS exposure.

## Review

Methods

This systematic review was conducted to evaluate the impact of passive smoking on child health, specifically focusing on respiratory conditions, behavioral problems, cognitive impairment, growth retardation, and immune system effects. Preferred Reporting Items for Systematic Reviews and Meta-Analyses (PRISMA) 2020 guidelines were followed to establish a comprehensive and transparent methodology for the review [9]. 

Search Strategy

A comprehensive literature search was conducted across multiple databases, including PubMed/MEDLINE (Medical Literature Analysis and Retrieval System Online), ScienceDirect, Europe PubMed Central (PMC), Cochrane Central Register of Controlled Trials (CENTRAL), Elton B. Stephens Company (EBSCO) Open Dissertations, and ClinicalTrials.gov. The search utilized a combination of keywords and MeSH (Medical Subject Headings) terms related to passive smoking, child health outcomes, and relevant interventions (Table 1).

**Table 1:** Search strategy CENTRAL: Cochrane Central Register of Controlled Trials; Europe PMC: Europe PubMed Central; EBSCO Open Dissertations: Elton B. Stephens Company Open Dissertations; MeSH: Medical Subject Headings; MEDLINE: Medical Literature Analysis and Retrieval System Online

Search strategy	Databases/ registers	Number of studies before/after filters	Filters applied
("Respiratory diseases"[All Fields] OR "Asthma"[All Fields] OR "Bronchitis"[All Fields] OR "Pneumonia"[All Fields] OR "respiratory infections"[All Fields] OR "sudden infant death syndrome"[All Fields] OR "SIDS"[All Fields] OR "Cognitive impairment"[All Fields] OR "behavioral problems"[All Fields] OR "low birth weight"[All Fields] OR "Growth retardation"[All Fields] OR "developmental delays"[All Fields] OR "otitis media"[All Fields] OR "ear infections"[All Fields] OR "hearing loss"[All Fields] OR "Cancer risk"[All Fields] OR "lung cancer"[All Fields] OR "immune system"[All Fields]) AND ("Passive smoking"[All Fields] OR "Secondhand smoke"[All Fields] OR "Involuntary smoking"[All Fields] OR "Environmental tobacco smoke"[All Fields] OR ("tobacco smoke pollution/adverse effects"[MeSH Terms] OR "tobacco smoke pollution/legislation and jurisprudence"[MeSH Terms] OR "tobacco smoke pollution/prevention and control"[MeSH Terms])) AND ("Children"[All Fields] OR "infants"[All Fields] OR "Adolescents"[All Fields] OR "pediatrics"[All Fields] OR "newborns"[All Fields] OR "Minors"[All Fields]) AND ("smoke free legislation"[All Fields] OR "Smoking cessation"[All Fields] OR "parental education"[All Fields] OR "prevention programs"[All Fields])	PubMed/MEDLINE	210/166	Humans, Full text, English
“Passive smoking” AND “Respiratory diseases” OR “behavioral problems” AND “Smoke-Free Legislation” AND “Children” OR "Pediatrics" AND ( "RCT" OR cohort OR "cross-sectional study" OR "observational study" OR "case-control" OR "clinical study" OR "Randomized clinical trial" ) NOT ( "Review" OR "Meta-analysis" )	EBSCO Open Dissertations	112/112	Dissertations
(“Passive smoking OR secondhand smoke OR involuntary smoking") AND ("Respiratory diseases OR respiratory infections OR sudden infant death syndrome OR behavioral problems " AND " children " AND "Smoke-free legislation "	Science Direct	4538/175	Research articles, Medicine and dentistry, English, Open access and open archive
((“Passive smoking") AND ("Respiratory diseases" OR "sudden infant death syndrome" OR "behavioral problems") AND (“Children") AND (“Smoke-free legislation")) AND (((SRC: MED OR SRC: PMC OR SRC: AGR OR SRC: CBA) NOT (PUB_TYPE: "Review"))) AND (HAS_FT: Y)	Europe PMC	35/26	Full text, Research articles
#1 ( "Passive smoking" ) OR ( "secondhand smoke" ) OR ( "involuntary smoke" ) OR ( "environmental tobacco smoke" ) #2 ( “Respiratory diseases” ) NEAR ( “Asthma” ) NEAR ( “Bronchitis” ) OR ( “Pneumonia” ) OR ( “respiratory infections” ) OR ( “sudden infant death syndrome” ) OR ( “SIDS” ) OR ( “Cognitive impairment” ) OR ( “behavioral problems” ) OR ( “low birth weight” ) OR ( “Growth retardation” ) OR ( “developmental delays” ) OR ( “otitis media” ) NEAR ( “ear infections” ) OR ( “hearing loss” ) OR ( “Cancer risk” ) OR ( “lung cancer” ) OR ( “immune system” ) #3 ( “Children” ) OR ( “infants” ) OR ( “Adolescents” ) NEXT ( “pediatrics” ) OR ( “newborns” ) OR ( “Minors” ) #4 ( “Smoke-Free Legislation” ) OR ( “Smoking cessation” ) OR ( “parental education” ) OR ( “prevention programs” ) #5 #1 AND #2 AND #3 #6 #5 AND #4	CENTRAL	25/12	Only clinical trials, excluding reviews, protocols, editorials, clinical answers
"Passive Smoking" | “Smoke-Free Legislation” OR “Smoking cessation” OR “parental education” OR “prevention programs” | Child (birth - 17) | Interventional, Observational studies | Studies with results	Clinical trials.gov Register	36/2	Age <18 years, Interventional, Observational With results

Inclusion and Exclusion Criteria for Study Selection

The eligibility criteria for this study were established to ensure the selection of relevant populations, interventions, outcomes, and study designs while excluding those that do not align with the research objectives (Table 2).

**Table 2:** Inclusion and exclusion criteria for study selection SHS: secondhand smoke; RCT: randomized controlled trials

	Inclusion criteria	Exclusion criteria
Population	Children<18 years. Children with documented or reported exposure to SHS at home, school, or other environments. Parents or primary caregivers who smoke and have at least one child aged < 18 years. Parents or caregivers who have participated in or are currently involved in smoking cessation programs.	Children aged 18 years of age and above. Children with no reported or documented exposure to SHS. Parents or caregivers who do not smoke. Parents or caregivers who have never attempted smoking cessation or have no exposure to smoke-free legislation.
Exposure/Intervention	Children exposed to SHS as reported by parents, caregivers, or through medical records. Parents or caregivers who smoke and are exposed to or aware of smoke-free legislation. Families who reside in places or regions where smoke-free laws are in effect.	Studies or cases where the source of smoke exposure is primarily from non-tobacco sources (e.g., exposure to smoke from other substances). Families living in areas without any smoke-free legislation or where such legislation is not enforced.
Outcome Measures	Health outcomes in children related to SHS exposure, including respiratory conditions, behavioral and cognitive issues, and growth or developmental impacts. Parental smoking behavior, including cessation attempts, success rates, and responsiveness to smoking cessation messages. Legislation banning smoking's impact on parental smoking habits and children's long-term health.	Studies that do not assess the health impact of SHS on children. Studies focusing on adult-only populations or outcomes unrelated to child health or parental smoking behavior. Interventions not related to smoking cessation or smoke-free legislation.
Study Design	Observational study, Clinical trials, and RCT	Editorials,Opinion pieces, Abstracts without full text, Reviews, and Uncompleted studies
Language	Studies published in the English language.	Studies published in languages other than English.
Year of Publication	Not specific	Specific time frame

Data Collection and Analysis

Data were extracted from the included studies using a standardized form. Extracted data included study characteristics, participant demographics, exposure details, health outcomes, and relevant findings. A narrative synthesis of the findings was performed, focusing on the impact of passive smoking on the specified health outcomes in children.

Screening and Quality Assessment

The initial screening was conducted by a single reviewer who used Rayyan [10] to evaluate the titles and abstracts of each article. Based on the assessment, the articles were categorized for potential inclusion or exclusion. Next, two independent reviewers performed a thorough full-text screening of the articles flagged for further review; they applied predefined inclusion and exclusion criteria to ensure the relevance and quality of the studies. In cases where the two reviewers disagreed on whether an article should be included, a third reviewer stepped in to resolve the conflict and make the final decision. 

Although we included various study designs, the final results yielded observational studies; thus, we employed the Newcastle-Ottawa Scale (NOS) for quality assessment [11]. Based on the overall risk of bias assessment, each study was classified as having a low, moderate, or high risk of bias.

Results

**Figure 1:**
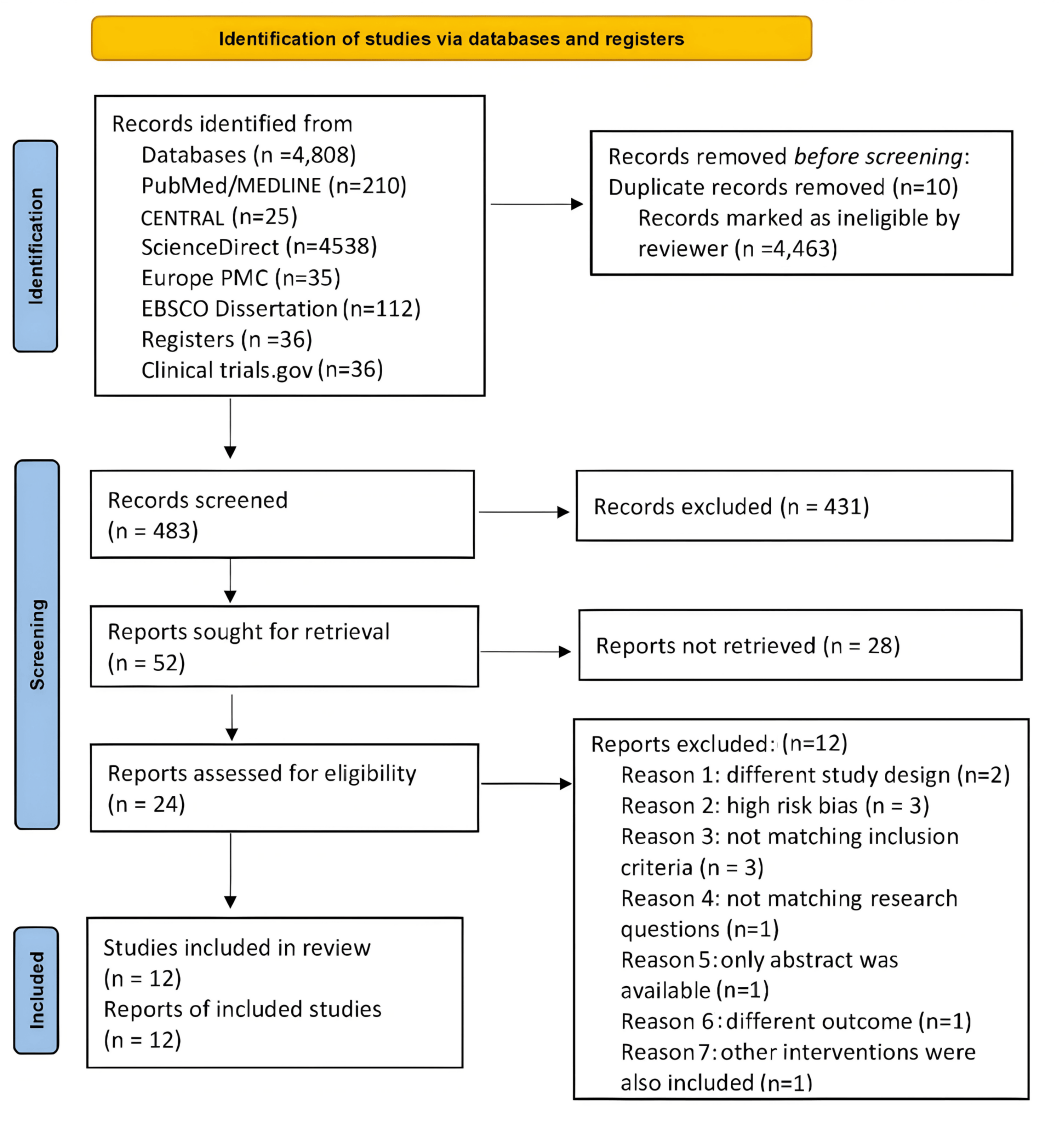
PRISMA flow diagram PRISMA: Preferred Reporting Items for Systematic Reviews and Meta-Analyses; CENTRAL: Cochrane Central Register of Controlled Trials; Europe PMC: Europe PubMed Central; EBSCO Open Dissertations: Elton B. Stephens Company Open Dissertations; MEDLINE: Medical Literature Analysis and Retrieval System Online

A thorough search strategy generated 4956 initial records from multiple databases. After applying predefined inclusion and exclusion criteria, 483 studies were deemed suitable for screening. From this pool, 52 studies were selected for retrieval, and 24 were subsequently assessed for eligibility. Ultimately, 12 studies met all criteria for inclusion in the review. The PRISMA flow diagram (Figure 1) demonstrates the screening process done to include the 12 studies for review. 

*The Results of Quality Assessment* 

Out of all observational studies, several were excluded due to moderate to high risk of bias. Ratajczak et al. presented a cross-sectional study with a high risk of bias, primarily due to insufficient detail on non-included subjects and a lack of comprehensive adjustments for confounding factors [12]. Despite having a solid sample size and structured assessment methods, it received a score of six out of nine, leading to its exclusion from future analysis. Similarly, Chastang et al. had a low response rate of 54.3% and relied on parent-reported questionnaires, raising concerns about representativeness and potential bias, resulting in a score of six [13]. Bleakley et al. scored five out of nine due to issues with non-response rates and measurement validity [14], also leading to its exclusion from future analysis (Table 3).

**Table 3:** Quality assessment of observational studies: Newcastle-Ottawa Scale scores Note: For cohort studies, a maximum of four stars ( * ) could be awarded for selection, two for comparability, and three for outcome domains. The total score ranges from zero to nine. For cohort studies, studies with scores ≥ 7-9, 4-6, and <4 are considered low, intermediate, and high risk, respectively. For cross-sectional studies, a maximum of five stars ( * ) could be awarded for selection, one for comparability, and three for outcome domains. The total score ranges from zero to eight. For cross-sectional studies: Studies with scores ≥ 7-9, 4-6, and <4 are considered low, intermediate, and high risk, respectively.

Study/Year	Selection	Comparability	Outcome	Overall
Chastang et al., 2015 [13]	****	*	*	******
Jenssen et al., 2021 [15]	****	**	***	*********
Bleakley et al., 2014 [14]	**	*	**	*****
Millett et al., 2013 [16]	****	**	***	*********
Cornelius et al., 2007 [17]	****	**	***	*********
Polus et al., 2021 [18]	****	**	***	*********
Anderka et al., 2010 [19]	****	**	***	*********
Been et al., 2015 [20 ]	****	**	***	*********
Sontag et al., 2020 [21]	****	*	**	*******
Ratajczak et al., 2018 [12 ]	***	*	**	******
Hawkins et al., 2016 [22]	****	**	***	*********
Ko et al., 2013 [23]	****	**	***	*********
Continente et al., 2019 [24]	*****	*	**	********
Rado et al., 2022 [25 ]	****	**	***	*********
Jacob et al., 2020 [26 ]	*****	*	**	********

The Characteristics of Included Studies

This systematic review includes 12 observational studies that focus on the impact of SHS exposure on children's health outcomes. The populations studied vary widely, encompassing groups such as parent smokers of pediatric patients, postpartum women, and children of various age ranges.

The aim of these studies includes understanding parental preferences for smoking cessation messages, assessing the effects of smoke-free legislation on childhood asthma admissions, and examining the relationship between prenatal tobacco exposure (PTE) and child behavior. Other studies investigated the impacts of smoke-free laws on pregnancy outcomes, respiratory infections, and the overall health of children exposed to SHS.

The exposures or interventions examined include parental smoking during pregnancy, smoke-free legislation enacted in different regions, and SHS exposure in homes. Key findings reveal that maternal smoking is linked to adverse birth outcomes, including LBW and preterm birth. At the same time, smoke-free legislation has shown varying effects on hospital admissions for respiratory conditions and asthma diagnoses. Additionally, studies indicate a need for tailored smoking cessation strategies to effectively engage different parent groups and improve communication practices among healthcare providers regarding smoking risks. More details are shown in Table 4.

**Table 4:** Summary of included studies ETS: environmental tobacco smoke; RTI: respiratory tract infection; SHS: secondhand smoke; SIDS: sudden infant death syndrome; PTE: prenatal tobacco exposure; LBW: low birth weight; SGA: small for gestational age; LRTI: lower respiratory tract infections; URTI: upper respiratory tract infection; OM: otitis media; CPRD: Clinical Practice Research Datalink; ED: emergency department; MIC: middle-income countries; aIRR: adjusted incidence rate ratios

Study Author/Year	Population	Exposure/intervention	Aim of Study	Key Findings
Jenssen et al., 2021 [15]	The study focused on 180 parent smokers of pediatric patients at four pediatric primary care sites.	Parents were asked to evaluate 26 different smoking cessation messages in a study.	To understand parents' preferences for smoking cessation messages to help clinicians tailor interventions that could increase the likelihood of quitting.	The analysis identified three groups of parents with distinct message preferences: In the first group, 51% of parents gave the effects of smoking on children a top priority. Group 2 (35%): Favored gain-framed messages emphasizing benefits. Group 3 (14%): Preferred messages focusing on the financial impact of smoking. Parents in Group 2 were prone to have a child over the age of six who had asthma and possessed inadequate health literacy. Tailoring smoking cessation messages to each group could improve motivational strategies and support precision medicine.
Millett et al., 2013 [16]	All children aged 14 years or younger who were admitted to the ED with a primary diagnosis of asthma.	Enacted smoke-free legislation in England in July 2007.	To determine how hospital admission for childhood asthma is affected by smoke-free legislation.	The admission rate changed significantly over a short period of time, by -8.9% (adjusted rate ratio 0.91; 95% CI: 0.89-0.93), and over time, by -3.4% year (adjusted rate ratio 0.97; 95% CI: 0.96-0.98).
Cornelius et al., 2007 [17]	All pregnant adolescents (aged 12-18) who attended the prenatal clinic.	PTE	To examine the relationship between PTE and child behavior.	PTE predicted significantly increased offspring activity, impulsivity, aggression, externalizing, and total behavior problems.
Polus et al., 2021 [18]	Newborns	Bavarian smoke-free legislation.	To examine the impact of the Bavarian smoke-free legislation on pregnancy outcomes.	There were ambiguous impacts for most outcomes. Significant statistical decreases were reported for both rapid and progressive reductions in very preterm deliveries. After the legislation was put into effect, there was an abrupt 10.4% drop. (95%CI − 15.8, − 4.6%; p = 0.0006) and a gradual decline of 0.5% (95%CI − 0.7, − 0.2%, p = 0.0010) in very preterm births.
Anderka et al., 2010 [19]	4,667 mothers of non-malformed control infants (National Birth Defects Prevention Study, 1997-2003)	Maternal smoking and ETS; assessed pharmacotherapy use for cessation	Describe tobacco exposure; identify risk factors; assess pharmacotherapy use; evaluate birth outcomes by smoking status.	20.6% reported smoking; 30.0% reported ETS exposure. 53.3% of smokers quit during pregnancy (74% in the first trimester). Only 2.1% used pharmacotherapy. LBW and preterm delivery rates are highest among continuing smokers.
Been et al., 2015 [20]	Children aged 12 years old and under whose data was in the CPRD database between 1997 and 2012.	Smoke-free legislation	To determine the effect of smoke-free legislation on the incidence of RTIs and new diagnoses of wheezing or asthma in children.	It was not associated with a statistically significant change in the number of new wheezing/asthma diagnoses in GP practices: IRR 0.94 (95% CI 0.81–1.09, p = 0.412). No statistically significant difference in the incidence of RTIs in the GP practice after smoke-free legislation was introduced (IRR 0.95 (95% CI 0.84–1.06), p = 0.399). Likewise, the legislation had no statistically significant impact on either the incidence of URTIs (IRR 0.95 (95% CI 0.86–1.06), p = 0.401) or LRTI episodes (IRR 0.96 (95% CI 0.81–1.15), p = 0.678).
Sontag et al., 2020 [21]	316 ob/gyns were surveyed about smoking cessation practices	Assessment of communication practices regarding SHS, SIDS, and smoking cessation	To evaluate current communication practices about SHS/SIDS risks and identify areas for improvement	(55.3%) spent 1-4 minutes discussing risks/cessation; 30% did not use supplemental materials. 51.9% felt sufficiently informed about SHS/SIDS risks. Improved communication may result from additional discussion time and use of materials, and immediate postpartum discussions could help prevent smoker relapse.
Hawkins et al., 2016 [22]	children aged 0–17 years who presented to outpatient ED in Massachusetts (2001–2010), New Hampshire (2001–2009), and Vermont (2002–2010).	Smoke-free legislation	To evaluate the effect of smoke-free legislation on children’s health	Among 10–17-year-olds, state smoke-free legislation was associated with a 12% reduction in ED visits for asthma (aIRR 0.88; 95% CI 0.83, 0.95), an 8% reduction for ear infections (0.92; 0.88, 0.97), and a 9% reduction for URTI (0.91; 0.87, 0.95). It has found that legislation to reduce smoking has led to a 10% reduction in ED visits for LRTI in children aged 0 to 4 years (0.90; 0.85, 0.94 ) and an 11% reduction for 10–17-year-olds (0.89; 0.84, 0.95). In general 8% decrease in LRTI-related ED visits (0.92; 0.87, 0.96) after the legislation's enactment.
Ko et al., 2013 [23]	21,248 postpartum women from the Taiwan Birth Cohort Study (June 2005-July 2006)	Assessment of parental smoking during preconception and throughout pregnancy stages.	To investigate the association between parental smoking and birth weight, LBW, SGA, and preterm birth	Maternal smoking was linked to decreased birth weight and higher incidences of LBW, SGA, and preterm birth, especially with >20 cigarettes/day. Paternal smoking showed no significant association.
Continente et al., 2019 [24]	2411 Spanish households with children younger than 12 years old	SHS	To determine the number of hospital admissions and incident cases associated with SHS exposure in the home for LRI, OM, and asthma.	Approximately 140,000 new cases of respiratory illnesses were caused by exposure to SHS. ∼3000 hospital admissions due to respiratory diseases were attributable to SHS exposure. 9%–13% of incident cases and hospital admissions were attributable to SHS exposure.
Rado et al., 2022 [25]	Neonates and Infants	Smoke-free legislation	To evaluate the impact on newborn mortality and infant mortality in all MICs that implemented this legislation and had data on the outcomes for a minimum of three years following the intervention.	Neonatal mortality decreased by 1.63% a year, whereas infant mortality decreased by 1.33% annually.
Jacob et al., 2020 [26]	37,505 adolescents aged 12–15 years who never smoked	SHS	To examine the correlation between exposure to SHS and the manifestation of depressive symptoms.	A study has found a clear connection between exposure to SHS and the experience of depressive symptoms. Specifically, compared to those who were not exposed to SHS, individuals exposed to SHS for 1-2 days had an OR of 1.06 (95% [CI] = 0.95, 1.18), those exposed for 3-6 days had an OR of 1.38 (95% CI = 1.20, 1.58), and those exposed for 7 days had an OR of 1.63 (95% CI = 1.44, 1.86).

Discussion

This systematic review adhered to the PRISMA 2020 guidelines [9] and aimed to assess the impact of passive smoking on children’s health, with a particular focus on respiratory conditions, behavioral issues, cognitive impairment, growth retardation, and effects on the immune system. The review also evaluated the effectiveness of smoke-free legislation in reducing these harmful outcomes. Additionally, it explored parental preferences for smoking cessation messages and the role of healthcare communication in minimizing the risks of SHS exposure. A comprehensive search identified and synthesized findings from 12 relevant studies. The studies reviewed highlight the significant impact of both passive smoking and smoke-free legislation on various health outcomes in children.

Behavioral and Cognitive Outcomes

The study by Cornelius et al. highlighted the detrimental effects of PTE on child behavior, including increased activity, impulsivity, and aggression [17]. These outcomes agree with the findings of Jacob et al., who found a link between adolescent depression symptoms and SHS exposure [26]. The dose-response relationship observed in their study further emphasizes the need for interventions to reduce SHS exposure to protect children’s mental health.

Respiratory Health and Hospital Admissions

Smoke-free laws did not significantly affect the incidence of RTIs or new asthma diagnoses, according to Been et al. [20]. However, Continente et al. reported that a significant number of hospital admissions for respiratory illnesses in children could be attributed to SHS exposure in the home [24]. Rado et al. investigated MICs neonatal and infant mortality following smoke-free laws [25]. The study found that such legislation led to annual decreases in both neonatal and infant mortality rates, underscoring the importance of smoke-free policies as a global public health strategy. The reduction in mortality rates highlights the life-saving potential of legislative interventions in reducing exposure to tobacco smoke during critical periods of infant development.

These findings suggest that while smoke-free legislation may reduce certain health risks, SHS exposure in domestic settings remains a critical issue that needs to be addressed.

Birth Outcomes and Infant Health

Studies by Anderka et al. [19] and Ko et al. [23] provide evidence of the adverse effects of maternal smoking and SHS exposure on birth outcomes, including LBW, SGA infants, and preterm births. Anderka et al. also noted the low use of pharmacotherapy for smoking cessation among pregnant women, highlighting a potential area for intervention to improve maternal and infant health outcomes [19].

Impact of Smoke-Free Legislation 

Several studies have assessed the effectiveness of smoke-free legislation in reducing health risks associated with SHS exposure. The implementation of smoke-free laws in England resulted in a noteworthy decrease in hospital admissions for childhood asthma, according to Millett et al. [16]. Similarly, Polus et al. observed a decrease in very preterm births after the introduction of smoke-free policies in Bavaria [18]. Hawkins et al. showed that children in states with smoke-free legislation had fewer ED visits for asthma, ear infections, and respiratory illnesses [22]. These findings collectively underscore the positive impact of such policies on public health.

*Parental Preferences and Communication* 

The research by Jenssen et al. demonstrates the importance of tailoring smoking cessation messages based on parental preferences [15]. Their study identified three distinct groups of parents with varying message preferences, suggesting that personalized communication strategies could enhance the effectiveness of smoking cessation efforts. Similarly, Sontag et al. emphasized the need for improved communication practices among obstetrician-gynecologists regarding the risks of SHS and SIDS [21]. The study revealed that a significant proportion of clinicians spend minimal time discussing these risks, indicating a gap that could be addressed through enhanced education and communication materials.

Comparison With Other Evidence

This review highlights the critical need for effective smoke-free legislation to mitigate the adverse health effects of passive smoking on children. The findings from our review are supported by other meta-analyses, including those by Rado et al. [27] and Nanninga et al. [28] which confirm the significant reductions in children's exposure to tobacco smoke following the implementation of smoke-free policies. Rado et al. found that smoke-free car policies significantly decreased TSE in children, suggesting potential respiratory health benefits [27], while Nanninga et al. reported that public smoking bans led to a notable reduction in SHS exposure in children's homes (RR = 0.72; 95%CI = 0.62-0.83) [ 28]. Together, these studies reinforce the importance of comprehensive smoke-free policies as a vital public health strategy to protect children's health from the harmful effects of tobacco smoke. 

*Strengths and Limitations of the Systematic Review* 

The review demonstrates several strengths, including a comprehensive literature search that utilized multiple databases, ensuring a wide range of relevant studies were considered. This wide-ranging methodology improves the findings' dependability. Additionally, the included studies represent diverse populations, encompassing various age groups and geographic locations, which improves the generalizability of the results. The review's focus on multiple health outcomes related to passive smoking, such as respiratory, behavioral, cognitive, and growth-related issues, provides a holistic understanding of the issue. Furthermore, adherence to PRISMA guidelines promotes transparency and reproducibility in the methodology.

However, there are notable limitations. Variability in methodologies for assessing exposure to SHS may lead to inconsistencies in results across studies. Many studies primarily focused on short-term outcomes, which limits insights into the long-term effects of passive smoking on children's health. Additionally, the review's restriction to studies published in English may introduce language and publication bias, potentially excluding relevant research from other linguistic contexts.

Clinical Implications

The findings of this review highlight the urgent need for healthcare providers to prioritize discussions about the risks associated with passive smoking with parents and caregivers. It is essential to develop tailored smoking cessation interventions based on parental preferences to enhance engagement and effectiveness. Furthermore, the implementation and enforcement of smoke-free legislation are critical for protecting children from the harmful effects of SHS. Such measures safeguard vulnerable groups' well-being and greatly enhance public health outcomes.

Future Research

Future research should focus on conducting longitudinal studies to better understand the chronic effects of passive smoking on children's health outcomes. There is also a need for standardized methods for assessing exposure to SHS, which would improve the comparability of findings across studies. Evaluating the effectiveness of tailored smoking cessation programs and smoke-free policies in diverse populations is crucial for informing public health strategies. Additionally, exploring the biological mechanisms through which passive smoking affects child health could lead to more targeted interventions. Furthermore, expanding research to include non-English studies and populations from various socio-economic backgrounds will enhance the understanding of the impact of passive smoking on a global scale. Finally, a more in-depth exploration of regional variations in smoke-free legislation and their impact on health outcomes is warranted, as these factors can significantly influence the effectiveness of public health interventions.

## Conclusions

This systematic review highlights the significant adverse health effects of passive smoking on children, including respiratory illnesses, behavioral issues, and developmental challenges. The evidence underscores the urgent need for comprehensive smoke-free legislation and tailored smoking cessation interventions that consider parental preferences and communication strategies. While smoke-free laws have shown promise in reducing exposure and improving health outcomes, variability in effectiveness suggests that ongoing evaluation and adaptation are necessary. Longitudinal studies should be the main emphasis of the next studies to evaluate the efficacy of therapies and the long-term effects of SHS exposure. Protecting children from the harmful effects of passive smoking is imperative for promoting their health and well-being, necessitating coordinated efforts from healthcare providers, policymakers, and families.
